# Understanding Variation in Sets of N-of-1 Trials

**DOI:** 10.1371/journal.pone.0167167

**Published:** 2016-12-01

**Authors:** Artur Araujo, Steven Julious, Stephen Senn

**Affiliations:** 1 Competence Center for Methodology and Statistics, Luxembourg Institute of Health, Strassen, Luxembourg; 2 Medical Statistics Group, School of Health and Related Research, University of Sheffield, Sheffield, United Kingdom; National Taiwan University, TAIWAN

## Abstract

A recent paper in this journal by Chen and Chen has used computer simulations to examine a number of approaches to analysing sets of n-of-1 trials. We have examined such designs using a more theoretical approach based on considering the purpose of analysis and the structure as regards randomisation that the design uses. We show that different purposes require different analyses and that these in turn may produce quite different results. Our approach to incorporating the randomisation employed when the purpose is to test a null hypothesis of strict equality of the treatment makes use of Nelder’s theory of general balance. However, where the purpose is to make inferences about the effects for individual patients, we show that a mixed model is needed. There are strong parallels to the difference between fixed and random effects meta-analyses and these are discussed.

## Introduction

N-of-1 trials are studies in which the effects of treatment are studied by following an individual patient over time with the treatments given being varied (randomised) from period to period. Thus, different treatments will be tried on different occasions by a given patient according to a randomisation scheme determined by the ‘trialist’, who may be the patient’s treating physician, in an attempt to improve treatment for that patient.

N-of-1 trials have a long history that predates the modern term—which was proposed in the 1980s by an influential group of researchers at McMaster University in Canada, including Gordon Guyatt and David Sackett[1–3]. It has also been the case, of course, that efficacy has been accepted as proven on occasion by single or at least very few successful cases. The rabies vaccine of Pasteur or the early studies of penicillin are cases in point. However, in this article we shall be concerned with designs in which at least two treatments are compared and where the treatments are compared within patient by switching the treatment given from occasion to occasion. We are further interested in the situation where more than one patient is treated.

An early example of n-of-1 trials was provided by Cushny and Peebles[4], who in their famous study of optical isomerism, gave three possible soporifics on multiple different occasions with intervening control nights to inmates of the "Insane Asylum at Kalamazoo", in order to examine the effect of treatment on ‘hours of sleep gained’[5]. These data were later used by Student in his famous t-test paper[6].

Cushny and Peebles did not use randomisation but this was advocated in 1930 by RA Fisher in *The Design of Experiments*[7], albeit not in a medical context, in a famous account of his testing of the ability of Rothamsted algologist Muriel Bristol to distinguish cups of tea by taste according to the order in which milk had been poured[8].

Perhaps the most important forerunner in the medical field is Paul Martini’s innovative but at the time neglected monograph of 1932[9, 10]. In this work he stressed the importance of control, blinding, replication and statistical analysis in determining causation in medicine and advocated the approach of repeatedly testing individual patients where possible.

A more modern example is provided by the trial of nicotine chewing gum in ulcerative colitis carried out by Lashner et al[11]. Seven subjects were allocated to two two-week periods of treatment with nicotine and two with placebo. Measurement was limited to the latter part of each period to minimise the possible effect of carry-over and a number of scales were used to measure outcome. For a recent review, advocacy and recommendation of the design, we refer the reader to Duan et al[12].

N-of-1 trials can be encountered under various names. In the social science literature, they go by the name of *single-case* or *single-subject* designs and are quite common. There are, for example, at least four monographs [13–16] devoted to statistical approaches to analysing such trials in psychology or sociology. In fact, RA Fisher describes the tea-tasting trial as a ‘psycho-physical experiment’[7] (p11). However, in this paper we shall restrict our attention to the medical application of such trials and, in particular, to methods of analysing the results that use either randomisation theory or mixed models, and in particular meta-analysis, all of which are a very common approaches in medical statistics.

One of the attractions of n-of-1 trials is that the fact that patients act as their own control means that results can be obtained using fewer patients. There are two reasons for this. First the number of observations per patient is increased. Second, a source of variation, the ‘main effect’ of patients can be eliminated. This means that for rare diseases they can be an attractive option. Generally, it is necessary that the condition being treated is long-term and that the effects of treatment are reversible as regards the particular outcome being measured, otherwise repeated switches of treatment given in order to compare them, is either not a practical possible or likely to deceive.

A referee has rightly pointed out to us that n-of-1 trials have found wide-spread application in examining established therapies with a view to personalising their use. We agree and, indeed, more than 20 years ago one of us wrote an editorial arguing that this was their most useful application[17]. However, our participation in IDEAL (Integrated DEsign and AnaLysis of small population group trials) a European Union funded FP7 project on statistical approaches to studying rare diseases, has led to our evaluating n-of-1 trials as a possible primary means to investigate the effects of treatment. Both these purposes are reflected in this paper.

Thus, as discussed above, a reason to undertake n-of-1 trials could be the efficiency in studying treatments. However, n-of-1 trials are also useful for establishing the personal component of response to treatment. A common notion in the medical literature is that this will be done individually *ab initio* for each patient. An n-of-1 protocol thus becomes a means of establishing for a given patient, using only results from that patient, what works best for them.

The information from one patient only might also be useful as the basis for indicating whether a treatment works at all, the idea being that if it works in at least one patient it may work in others. This would generally require rather a long series of episodes of treatment and possibly complex modelling. There has been some work on such series, a key paper is that of Rochon[18]. The emphasis in this paper is not on analysing individual trials but instead on approaches to analysing series of n-of-1 trials carried out in a number of patients.

Where we have data on many patients an interesting challenge is to combine results from a given patient with those from other patients to provide better prediction of effects for the individual patient. The basic idea is simply explained in terms of two extremes. First, if we have lots of information from many previous patients but do not have the possibility of experimenting on the current one but simply have to make a recommendation, then the estimated treatment effect, based on all the results of others, will be what we should use. On the other hand, given an extremely long ‘n-of-1’ series during which two treatments have been compared in one patient, the experience of others is of little relevance. In the intermediate case, with a little information on the current patient, some weighted average of the two extremes (only the results of others versus only the results of this patient) may perform better. This is what ‘mixed models’ can produce via so-called ‘shrinkage estimators’[19].

A further use is to establish the existence of treatment-by-patient interaction. A set of n-of-1 trials permits treatment-by-patient interaction, which is otherwise confounded with other sources of variation, to be identifiable. Whether such an interaction is substantial or negligible can be an indication as to what extent it is worth taking up the challenge to find ways of personalising medicine based on covariate information (for example, genetic markers)[20–22].

In this paper we shall mainly deal with the analysis of n-of-1 trials, although, inevitably, some matters of design will have to be touched on, since design affects analysis. We restrict our attention to the situation where a series of n-of-1 trials are performed according to a common protocol. Important papers discussing the analysis of such cases are those of Zucker and colleagues[23, 24]. From another point of view, such a series taken together, forms a cross-over trial [25, 26], albeit one in which the same treatment is given more than once to each patient. Such designs are sometimes discussed in the literature of cross-over trials but usually in the context of providing means of estimating carry-over effects rather than individualising treatment. See the third edition of Jones and Kenward for some illustrations of this[27].

We propose to examine a number of methods including four that were proposed in an informative paper by Chen and Chen[28] in their investigation of approaches to analysing a series of n-of-1 trials. To these four methods we shall add a fifth, the summary measures approach. However, our main contribution will be to provide a framework in which analysis is linked to design and purpose. We shall add estimation, the production of valid confidence intervals and shrinkage estimates, to the purpose of hypothesis testing that Chen and Chen considered. We shall show that these other purposes lead to an alternative perspective on suitable techniques. We will also make a comparison to meta-analysis and the framework that we provide will additionally illuminate a long-standing controversy between the use of fixed effect and random effects.

Following Chen and Chen[28] we shall restrict our attention to continuous measures. Although binary and count data can also be important their inclusion in this paper would make it long. We plan to revisit these data in a further paper. However, we will make some brief comments on approaches to analysing such data at the end of this paper

The outline of the paper is as follows. In section two we shall present the findings of Chen and Chen[28] briefly but also introduce one further approach. In section three we shall then consider carefully a set of n-of-1 trials in terms of their block structure as understood by what might be termed ‘The Rothamsted School’ of analysis of variance associated with statisticians such as Fisher[29–31], Yates[32], Nelder[33, 34] and Bailey[35, 36]. This school regards the following as being key to establishing appropriate approaches to testing hypotheses: 1) identification of sources of variation in the experimental material 2) identification of ‘blocks’ to control such variation 3) method of randomisation used.

In the fourth section we shall introduce a model that can be used for simulation and analysis and move beyond hypothesis testing to cover estimation and prediction, both for groups and individuals, and present some theoretical results and in the fifth we shall confirm and extend these using simulation. In the sixth section we shall give a simple simulated example to illustrate approaches to analysis. We shall discuss our findings in the seventh section and in the eighth present our conclusions and recommendations.

Before proceeding, however, we make two remarks about random effects. The first is a matter of terminology. Patients vary in their state of health. For example, some have higher blood pressure than others. If we choose to regard such variation as random in a model, this is an example of a random *main effect*. Patients may also vary in their *response* to treatment. For example, some might respond better to beta-blockers and others to ACE inhibitors. If we chose to model such variation in response as random, this is an example of a random *interaction*. A similar distinction is important in meta-analysis[37], where the fact that the treatment effect (difference between treatments) may vary (apparently randomly) from trial to trial is an example of a random treatment-by-trial interaction, whereas the fact that average values may vary from trial to trial (apparently randomly) is an example of a random main effect of trial. Clearly the main and interaction effects in trials can have their origins in main and random effects in patients.

The second point is to do with modelling treatment-by-patient interaction. It could be contended that testing such interactions is not particularly important. We agree. However, estimating the treatment-by-patient interaction is part of the process of fitting a mixed effect model, even if the fitting of the model happens in one step so, that this aspect is partly hidden. The connection to meta-analysis is of interest here. When one has sets of n-of-1 trials, since the set of repeated episodes of treatment for a given patient can be regarded as the ‘trial’.Indeed, Chen and Chen[28] use meta-analysis as one of their four methods of analysis. However, meta-analysis, shows the two faces of interaction. Cochran’s Q statistic can be compared to the degrees of freedom to form a test or can be used, as in the approach of DerSimonian and Laird[38], to estimate the random effect. The fact that we shall illustrate how treatment-by-patient interaction may be estimated does not imply that we regard testing such an effect as being more important than fitting a mixed model. In particular we reject as being completely inappropriate testing treatment-by-patient interaction as being a means of choosing between fixed and random effect models. As we shall explain, and as has been explained elsewhere in connection with meta.analysis[39] the difference between these two models is one of *purpose*.

## The Methods and Recommendations of the Chen and Chen Paper

The paper by Chen and Chen[19] considers designs in which two treatments A and B are compared. They assume *n* patients will either be treated in three or in four cycles of treatment. Each cycle consists of two pairs of what we shall call *occasions* and in each such pair of occasions A is given once and B is given once, the order being randomised. In what follows, we shall assume a general case in which the number of cycles per patient is *k*, where Chen and Chen[28] would either have *k* = 3 or 4 (we shall assume *k* is the same for all patients in any given design and that there are no missing data but will touch briefly on the more general case where this is not the case). There are thus *nk* cycles in total.The number of occasions is 2 per cycle and we shall refer to the product of cycle and occasions as *periods*. Thus there are 2*k* periods of observation per patient and if we refer to the sum total of all periods of observation over all patients as *episodes* of treatment we then have 2*nk* episodes in total.

Chen and Chen compare four approaches to analysis which we consider below along with an additional fifth approach. These are shown in *Table 1*.

**Table 1:** Methods for analysis n-of-1 trials.

Approach	Description
Method 1	A paired t-test approach using the differences calculated from the *nk* cycles and treating these as independent
Method 2	A mixed effects model for the difference, in which a common random effect is assumed for all cycles from the same patient. Although Chen and Chen do not state so explicitly, because the *nk* differences between treatments are the basic statistics that are modelled, rather than the 2*nk* original values, the random effect in question is a random interaction, the main effect already having been eliminated by differencing.
Method 3	A mixed effects model for the 2*nk* original values including a period effect to cover possible differences in average response for the 2*k* period simple carry-over effects a random main effect for the patient in addition to the treatment effect and a within patient error term.
Method 4	A meta-analysis approach. From our understanding of the paper by Chen and Chen[28] as they reference using the approach of DerSimonian and Laird[38], we have assumed the analysis approach is a random effects meta-analysis where the random effect in question is a random interaction.
Method 5	A fifth simple method, a summary measures approach[40], could be added to those above, and that is to reduce all measurements on the patients to a single contrast of the difference B-A. Thus, compared to the t-test, the unit of analysis becomes the patient rather than the cycle, there being *n* estimates of the treatment effect rather than *nk*. We shall also consider this alternative in due course below.

The key recommendations of Chen and Chen[28] can be summarised thus: if it may be assumed there is no carry-over effect, the matched t approach (method 1 above) is best. However, if it is assumed there is a carry-over, use the mixed model approach (method 3 above) on the original observations (although they found that the matched pairs t-test also performed well).

Of course, as in any method of investigation using simulations, the conclusions depend not only on the model used for analysis but also on the model used for simulation. For example, the methods that Chen and Chen use for *simulating* data allow for compound symmetry (with three possible parameter settings) or an auto-regressive structure (with two parameter settings) but the error models used do not allow for a random treatment-by-patient interaction However, two of the four methods of *estimation* not recommended can allow for such a random effect, namely methods 2 and 4.

Whether or not a random treatment-by-patient interaction should be allowed for is at least partly a question of purpose. If the purpose is strict hypothesis-testing, that is to say to establish whether there is any evidence of difference at all between treatments, then given the proposed randomisation, as we shall show below, method 1 is an acceptable approach (although it can be improved on). However, if the objective is to produce improved estimates for future patients by shrinking these estimates towards the overall mean, then method 1 cannot deliver this. The issue is very similar to that in meta-analysis with fixed effects approaches being fine for hypothesis testing but random effects approaches having some advantages for estimation[39].

A further relevant issue is the method of randomisation employed. Again, as we shall show below method 2 is not really consistent with the method of randomisation suggested.

In summary, the purpose and approach to randomisation, both of which are related to assumed structure of the experimental material and hence the models, need to be examined carefully to decide on the approach to analysis to be undertaken. In the next section we propose to examine these issues.

## Some theoretical considerations

We shall examine the design considered by Chen and Chen[28] in terms of the approach of The Rothamsted School, which is implemented in the algorithms that the statistical package GenStat® uses for analysis of variance and reflects John Nelder’s approach to linear models[41]. ANOVA models are built up from two fundamental components: the *block structure*, which reflects known variation in the experimental material and which has been reflected in the design and the *treatment structure*, which reflects the nature of the treatments applied to the experimental units.

In the work of Chen and Chen the assumption was of independent randomisation in cycles. This means that, there are 2^k^ possible random allocations that may be applied to any patient, so for the design in *k* = 3 cycles any patient may receive one of 8 sequences and for *k* = 4 cycles one of 16. However, an equal number of each treatment per patient could be attained by a more complete randomisation, one in which (2*k)*!/(*k*!*k*!) possible sequences could be used. This yields 20 such sequences for k = 3 and 70 such sequences for k = 4.

The restriction of forcing each treatment to appear once in each cycle makes sense if the purpose is to achieve some sort of local control for individual trend effects for each patient. This would be the case if one expected random fluctuations over time. It needs a variance term to cover this in any simulation. This in turn implies that the block structure is cycles nested within patients or *Patient/Cycle* to use the Wilkinson and Rogers (W&R) notation[42] and this together with a simple treatment structure of ‘Treatment’ in turn yields the valid analysis of variance decomposition according to randomisation theory using the general balance approach of Nelder[33, 34].

This can be easily implemented in GenStat®, which in advance of having any actual measurements for the design, will propose the skeleton analysis of variance. For a design with 12 patients and 3 cycles the result is shown in Table 2

**Table 2:** Degrees of freedom for a design with the block structure Patient/Cycle. The second column gives the degrees of freedom for treatment structure Treatment and the third with Treatment. Patient added. The case of 12 patients and 3 cycles is illustrated.

	Analysis	
	Without interaction	With interaction
Source of variation	df	df
Patient stratum	11	11
Patient.Cycle stratum	24	24
Patient.Cycle.*Units* stratum		
Treatment	1	1
Patient.Treatment	NA	11
Residual	35	24
Total	71	71

(Note that Patient/Cycle is equivalent to Patient + Patient.Cycle, that is to say the main effect of patient plus the interaction of patient and cycle, and that the statistical package R would use Patient:Cycle to represent the latter term, whereas SAS® would use Patient*Cycle.)

The important point in this is that the 1 degree of freedom for treatment has to be compared to the 35 degrees of freedom associated with the cycles. In such a design there are 12 x 3 = 36 cycles and this analysis is equivalent to the matched pairs method of Chen and Chen, since this would analyse 36 differences and remove one degree of freedom from the variance estimate for the overall mean. The general situation is shown in the second column of Table 3.

**Table 3:** Degrees of freedom for a design with the block structure Patient/Cycle. The second column gives the degrees of freedom for treatment structure Treatment and the third with Treatment. Patient added. The general case is illustrated.

	Analysis	
	Without interaction	With interaction
Source of variation	df	df
Patient stratum	n-1	n-1
Patient.Cycle stratum	n(k-1)	n(k-1)
Patient.Cycle.*Units* stratum		
Treatment	1	1
Patient.Treatment	NA	n-1
Residual	nk-1	n(k-1)
Total	2nk-1	2nk-1

Note there is a disturbing feature of the analysis and that is that residual degrees of freedom exceed the number of patients. In general, for such a design, we will have *nk*-1 residual degrees of freedom but only n-1 patients so that for *k* > 1, this will always be the case. Senn and Hildebrand[43] drew attention to this as a general feature of cross-over trials with more than two periods and, in fact, explicitly considered the case where two patients were repeatedly treated in many periods. They pointed out that the phenomenon of treatment-by-patient interaction might cause problems for inferences made from models using only one error term. (See [44] for a more recent recognition of this.)

If treatment-by-patient interaction is added to the treatment structure for the above analysis, then GenStat® delivers the decomposition shown in the third column of *Table 2*.

There are 11 degrees of freedom for interaction, the number arising as the product of 12–1 for patients and 2–1 for treatments. The analysis of variance will now remove these 11 degrees of freedom for interaction from the residual, An exactly analogous phenomenon arises in fixed effects meta-analysis where the trial-by-treatment interaction is removed from the overall estimate of variability because trial by trial the variance estimate is only local [39]. Applying the same philosophy here, and treating each of the 12 patients as a ‘trial’, we can see how this happens. We have 3 differences for each patient. If we estimate the variance for each patient, each such estimate has 2 degrees of freedom and there being 12 patents we end up with 24 in total.

The general case corresponding to this is given by the third column of *Table 3*, which considers a design with *n* patients in *k* cycles with an interaction fitted.

Having the treatment-by-patient interaction as fixed could be questioned. It might be fine for the *limited* purpose of establishing the average effect in the patients actually studied but in stating our uncertainty about patients in general it might seem to be more appropriate to treat the interaction as random. This can be done by imagining that each patient has some modification to the overall average treatment effect that is permanent for that patient but assigned at random. The natural extension of the matched pairs approach for dealing with this is then Method 2 of Chen and Chen[28].

In the simulations undertaken by Chen and Chen[28] there seems to be no allowance for treatment-by-patient interaction. Compound symmetry merely allows for two variance components: it allows for between-patient and within-patient variation. The former, however, is a main effect variance and not an interaction variance. It is also eliminated by subtraction due to the formation of the within-cycle difference for both method 1 and method 2. Chen and Chen also consider an auto-regressive process of order 1 (an AR1 process). (This allows for correlation between successive observations but in such a way that given the result of the most recent observation on a patient, those further back have no predictive value.) This does not allow for a treatment effect that varies randomly from patient to patient and in fact it does not even allow for a between-patient main effect, since as the interval between observations increases, the correlation declines without limit, yet a between-patient effect would impose a limit on the possible decline (A way to deal with this would be to assume that within patient errors alone follow an AR1 process). The compound symmetry models for the simulation also do not allow for variation from cycle to cycle above and beyond that which is simple within-patient variation. The AR1 models would go some way to reflecting this, albeit imperfectly.

Turning now to method 3, we see that this does not correspond to the method of randomisation, which is in cycles. If there is a between-cycle variance it will not be eliminated by such a model. The fact that the Chen and Chen simulation does not allow for a between-cycle variance places this method in a better light than would be the case if one were allowed for. However, the simulations of Chen and Chen allow for a common period effect. Using the W&R notation[42], the block structure is Patient*Period and this leads to the degrees of freedom decomposition given in the third column of Table 4. (Note that in the W&R notation A*B = *A*+*B*+*A*.*B*, where *A*.*B* is the interaction, so that A*B does not have the meaning it does in SAS® notation.) If compared with Table 2 it can be seen that the 24 degrees of freedom for cycles within patients have been pooled with the 35 for error but 5 have been removed for the period effect. This is inappropriate since the extra degrees of freedom for error have been removed by design from the variability associated with the treatment effect but not from the error term used to assess that variability.

**Table 4:** Degrees of freedom for a design with the block structure Patient*Period and the treatment structure Treatment. The case of 12 patients and 6 periods.

	Analysis	
	Without interaction	With interaction
Source of variation	df	df
Patient stratum	11	11
Patient.Cycle stratum	24	24
Patient.Cycle.*Units* stratum		
Treatment	1	1
Patient.Treatment	NA	11
Residual	35	24
Total	71	71

The degrees of freedom for the general case corresponding to Table 4 are given in Table 5. Here it is assumed that there are *m* periods where *m* is an even number and there are *n* patients where *n* is a multiple of *m*. The way in which randomisation would be carried out is then rather different to the design in cycles. One way of proceeding is as follows. First for every set of *m* patients one would choose an *m* x *m* Latin square at random. Such a design will allocate *m* pseudo-treatments for *m* patients in *m* periods. The rows and columns of the design chosen are then permuted at random. Finally, one half of the *m* pseudo-treatments are labelled as treatment A and the other half as treatment B.

**Table 5:** Degrees of freedom for a design with the block structure Patient*Period and the treatment structure Treatment. The case of n patients and m periods.

	Analysis	
	Without interaction	With interaction
Source of variation	df	df
Patient stratum	n-1	n-1
Patient.Cycle stratum	n(k-1)	n(k-1)
Patient.Cycle.*Units* stratum		
Treatment	1	1
Patient.Treatment	NA	n-1
Residual	nk-1	n(k-1)
Total	2nk-1	2nk-1

In summary, therefore, the method of randomisation assumed by Chen and Chen makes sense if

1. there is a main effect variation between patients
2. there is variation between cycles within patients.
  In addition, one of the purposes of n-of-1 trials is studying individual response and this implies
3. there is variation in such responses.

That is to say a treatment-by-patient interaction. In our opinion the parameters of the simulations of Chen and Chen do not take into account b) and c) and the results cannot be generalised to these situations.

In the next section we shall start by introducing a general model that is compatible with the randomisation scheme assumed and consider what this implies for methods of analysis.

### Models for simulation and analysis[45–49]

We start by building a model that reflects a simulation that would be compatible with the assumed block structure that corresponds to randomising within cycles within patients. The model can be written as

$$Y_{irs}=\lambda_{i}+\beta_{ir}+\varepsilon_{irs}+Z_{irs}\tau_{i}$$ (1)

where *Y*_*irs*_ is the measured outcome for occasion *s*,*s* = 1,2 of cycle *r*,*r* = 1,2…*k* for patient *i*,*i* = 1,2…*n* and *ε*_*irs*_ ∼ *N*(0,*σ*^2^), *β*_*ir*_ ∼ *N*(0,*γ*^2^), *λ*_*i*_ ∼ *N*(Λ,*ϕ*^2^) and *τ*_*i*_ ∼ *N*(T,*ψ*^2^) with $Z_{irs}=\frac{1}{2}\text{or}-\frac{1}{2}$ depending on whether the treatment allocated to that patient, in that cycle on that occasion, is A or B. All stochastic terms are assumed independent of each other. Here Λ is the expected value over all patients, cycles and occasions and is a sort of grand mean and T is the expected treatment effect over all patients. *λ*_*i*_ is a mean value for patient i. Note that the *τ*_*i*_ term is a random treatment effect for patient *i* but by setting the variance *ψ*^2^ equal to 0 we can make all such treatment effects identical. Similarly, and this is an important point we take up later, we can choose to regard our focus of interest as being not so much T, which is the expected value for a population of patients, as $\bar{\tau }=\sum_{i=1}^{n}\tau_{i}/n$, the average value *for the patients in this trial*. The two are only the same if *ψ*^2^ = 0 The former is a typical linear model formulation and the latter is associated with randomisation and causal inference. An excellent discussion of the distinction is given by Cox[50]. See also Senn[51]. For the moment, however, we stick with the linear model formulation, which agrees with the description of a simulation below.

The way this corresponds to a simulation is as follows:

1. Set all parameter values for the simulation.
2. Choose a patient i and simulate a value *λ*_*i*_ and another value *τ*_*i*_. These are to be retained and used until all values for that patient have been generated.
3. Generate a value *β*_*ir*_ for cycle r of patient i. This is to be retained and used for both occasions of that cycle of that patient’s course of treatments
4. Generate a random number *X*_*ir*_ from *U*(0,1) for that cycle.
5. Calculate $Z_{ir1}=sign\left(X_{ir}-\frac{1}{2}\right)/2,Z_{ir2}=-Z_{ir1}$
6. Generate a value *ε*_*irs*_ for occasion s of cycle r for patient i.
7. Calculate the response as the sum of the value of *λ*_*i*_ for that patient *β*_*ir*_ for that cycle, *ε*_*irs*_ for that occasion and the product of *τ*_*i*_ for that patient multiplied by *Z*_*irs*_ for that occasion.

Note that if we form paired differences from Eq (1) by subtracting the value for the first occasion in any cycle from the value for the second we obtain an equation of the form

$$d_{ir}=Y_{ir2}-Y_{ir1}=\left(Z_{ir2}-Z_{ir1}\right)\tau_{i}+\varepsilon_{ir2}-\varepsilon_{ir1}$$ (2)

Note that in Eq (2), (*Z*_*ir*2_ − *Z*_*ir*1_) is either equal to -1 or +1 depending on the random allocation since *d*_*ir*_ is what is sometimes referred to as a *period difference*. To create treatment differences (for example of the form B-A) we can divide through Eq (2) by (*Z*_*ir*2_ − *Z*_*ir*1_) to obtain

$$d_{ir}^{*}=\tau_{i}+\varepsilon_{irB}-\varepsilon_{irA}=T+\delta_{i}+\xi_{ir}$$ (3)

where *d*_*ir*_ is the observed treatment difference for patient *i* in cycle *r*, *δ*_*i*_ is the random difference in the treatment effect for patient i to the overall expected effect T, and is a random treatment-by-patient interaction with variance *ψ*^2^ and *ξ*_*ir*_ is a random error term with variance 2*σ*^2^. If Eq (3) is compared to Eq (1) of Chen and Chen (which is for their method 2) it will be found to be the same, making due allowance for differences in notation. Thus showing, as we claimed above, that their method 2, includes a patient-by- treatment interaction.

Provided that all patients receive the same number of cycles of treatment (which Chen and Chen assume), their method 1, will also produce exactly the same *estimate* for *any* data-set including, of course, any simulated one, as method 2. It thus follows that the observed variation from simulation to simulation will be identical whether their method 2 or their method 1 is used. The same is true for our proposed fifth method, the summary measures approach, and indeed, it would be true for yet another method, which is to use the mixed model corresponding to the simulation itself for estimation, in other words a model that corresponding to Eq (1).

The summary measures approach calculates the mean difference in outcome under B minus the mean difference in outcome under A per patient. In the literature this has been referred to as the *basic estimator* approach[25] and was proposed and developed to deal with the problem of degrees of freedom for error exceeding the number of patients. This is equivalent to calculating the mean patient by patient of the within-cycle treatment difference. A model for this statistic is thus

$${\bar{d}}_{i.}^{*}=\frac{\sum_{r=1}^{k}d_{ir}^{*}}{k}=T+\delta_{i}+\frac{\sum_{r=1}^{k}\xi_{ir}}{k}=T+\delta_{i}+{\bar{\xi }}_{i.}$$ (4)

Each such a summary statistic has a variance of *ψ*^2^ + 2*σ*^2^/*k* and, since the summary statistics are independent, the mean over the set of *n* patients will have variance

$$Var\left(\hat{T}\right)=\psi^{2}/n+2\sigma^{2}/\left(nk\right)$$ (5)

This is the true variance over all simulations of the estimate. Note that since all these methods produce the same estimate on every occasion, to the extent that we regard ourselves as estimating T, the mean treatment effect for the population of patients for which those studied could be regarded as a sample, they all have the same variance. (In fact expression (5) can be reached by starting with either of the models given by Eq (1) or (3).) The only question is ‘do they estimate it correctly?’.

The answer is that all do, except the matched pairs approach, which only does so if it is the case that *ψ*^2^ = 0. The summary measures approach does so directly by allowing the observed variation of the independent summary statistics to produce the estimate of the variation. Method 2 of Chen and Chen also does so correctly because it allows (implicitly) for a random treatment-by-patient interaction, as would a method based on applying a mixed model of the sort given by (1) to the original data. The procedure would be more laborious because it would estimate the various components of variation and add them back again. If the only purpose is to estimate T there is no advantage in this but for other purposes identifying components of variation and hence estimating individual effects it is valuable[52].

By estimating the variance of the treatment effect directly from the *n* treatment estimates themselves, the summary measures approach produces a sum of squares with expectation equal to (*n*−1)(*ψ*^2^ + 2*σ*^2^/*k*) and on division by first the degrees of freedom for this estimate and secondly the number of such statistics, n, the correct figure for the variance as given in (5) is produced. What the matched pairs procedure does is, starting from the differences, it adds the sums of squares for interaction to the residual sum of squares for the model fitting the interaction. The former has an expectation equal

$$k\left(n-1\right)\left(\psi^{2}+2\sigma^{2}/k\right)=\left(n-1\right)\left(k\psi^{2}+2\sigma^{2}\right)$$

and the latter has expectation equal to

$$n\left(k-1\right)2\sigma^{2}$$

Adding the two together we have an expectation for the total of

$$\left(n-1\right)k\psi^{2}+\left(nk-1\right)2\sigma^{2}$$

Since the degrees of freedom associated with this combined sum of squares are *nk*−1 the mean square has expectation equal to

$$\frac{n-1}{nk-1}k\psi^{2}+2\sigma^{2}$$

If we further divide this by the number of observations, to reflect what will happen in calculating the variance of the treatment estimate that the matched pairs approach will produce we get

$$\frac{n-1}{nk-1}\frac{k}{nk}\psi^{2}+\frac{2\sigma^{2}}{nk}=\frac{n-1}{nk-1}\frac{\psi^{2}}{n}+\frac{2\sigma^{2}}{nk}$$ (6)

See also Patterson and Jones[53] for a derivation.

If (6) is compared with (5) we will see that it is only the same when either k = 1, which corresponds to one cycle per patient or when *ψ*^2^ = 0 and there is no treatment-by-patient interaction. Otherwise, however, {(*n*−1)/(*nk*−1)}<1 and this means whereas the random patient by treatment interaction contributes a term *ψ*^2^/*n* to (5) only a fraction of this is contributed to (6). Thus (6) is an underestimate of the true variance. Hence standard errors will be too small and confidence intervals for the treatment effect T using the matched pairs approach will not have correct coverage (they will overstate the precision).

The same is not true if the object of the exercise is either to test the null hypothesis that the treatment effect is zero or to provide a statement for the treatment effect in these patients. As regards the latter it might be thought that this is of no interest. After all we have observed all the patients in the set of trials. However, this overlooks two facts. First, each patient could have been randomised to 2^k^ possible sequences of treatment but was only randomised to one. Second each patient could have been observed longer. Due to the natural variation from occasion to occasion captured by the term *ε*_*irs*_ either of these would have led to a different treatment estimate for that patient.

As regards the former, if we assert the null hypothesis that there is no treatment effect then there can also be no interaction and hence the matched pairs approach becomes a reasonable one. At first this may seem shocking but consider the case where we only have one patient so that n = 1. We can test the hypothesis that the treatments are identical by using that patient. Now if we study formula (5) and formula (6) by setting n = 1 we see that they give the same answer. The variance of the estimate for that patients becomes 2*σ*^2^/*k* and our estimate of that variance will have expectation 2*σ*^2^/*k* and we will have used *k*−1 degrees of freedom to estimate it. Each of the *n* patients gives us a legitimate attempt to test this theory and one approach is to combine these estimates. We can now see the value of the structured ANOVA approach and the relevance of *Table 3*. Combining the *n* estimates with their *k*−1 degrees of freedom leads to the ANOVA test allowing for an interaction and given in column 3. This goes even beyond the matched pairs t approach. That pools an interaction sum of squares with an error sums of squares, whereas this isolates the latter. This is essentially the approach of fixed effects meta-analysis, albeit using a pooled variance rather than estimating locally as is the case in meta-analysis.

It is possible that this approach could be more powerful than the matched pairs t. However, once a hint of efficacy has been provided by such a test, it is our recommendation that one should go beyond it to consider other matters, including estimation of the average effect for all patents and shrinkage estimates for future patients. This would require use of a random effects model. The reason we mention this alternative test here is because of our work on developing methods for rare diseases for which techniques for obtaining hints of efficacy using limited resources may be particularly valuable.

As stated earlier details as to exactly which method of meta-analysis Chen and Chen[28] used as their method 4 are not provided. From their formula (3) it would seem that they are proposing a locally standardised effect size such as was used by Glass in his original meta-analysis paper. This is usually employed when combining effects from trial to trial with different outcome measures and it would not be an obvious choice for n-of-1 trials. More usual would be to stick with the original un-standardised scale. In that case if the estimates from each patient are weighted equally and if the variance is estimated from the observed variation of those estimates from patient to patient, this is nothing less than the summary measures approach.

Note that the model we have introduced in Eq (1) is the one that corresponds to the randomisation scheme assumed by Chen and Chen[28]. Of their two approaches for analysis, both the paired t-test and the mixed model using within cycle differences will produce correct significance tests and the latter will also produce confidence intervals with correct coverage in the presence of an interaction. As already discussed, a meta-analytic approach could also work but this depends on how it is applied. Approach number three is rather different. This allows for a common period effect as opposed to randomisation within cycles, which would allow for local effects. The block structure as we have already implied is Patients*Periods. We have already criticised the AR1 structure assumed. There are two remaining problems, however. One is that no random treatment-by-patient effect is allowed for, yet the existence of such effects is one of the possible motivations for n-of-1 trials. The other is that the simple carry-over model used assumes that carry-over from A into B is the same as from A into A etc. As has been pointed out by Fleiss[54, 55] and Senn[56], this is not very realistic. In our opinion it is best not to include a carry-over term in the model but to make sure that the periods of observation for patients are long enough for the effects of previous treatments to have disappeared.

However, a mixed model can be adapted to apply to the case of a crossed structure. Eq (1) is then modified to look like this

$$Y_{ij}=\lambda_{i}+\pi_{j}+\varepsilon_{ij}+Z_{ij}\tau_{i}$$ (7)

Here *j* = 1⋯2*k* is an indicator for the period in which the patient is treated. For example, rather than randomising each patient to receive A or B in *k* = 3 cycles the patients could have been more completely randomised to 6 periods, perhaps using a system of Latin squares as outlined above. (In that case the number of patients recruited will be a multiple of 6.) Each period is given a period effect *π*_*j*_ but these are assumed to be the same for any patient. The random within-patient term is now *ε*_*ij*_. This is a possible mixed effects model that could apply to this alternative randomisation scheme.

Trivially, if (7) is assumed to be the data structure, then estimation using model (7) will be appropriate. A simple alternative is to use the basic estimator approach of Senn[25]. This constructs a summary measure for each patient but estimates the variation of these not across the design as a whole but first separately within each sequence. In this way the period effect, which has been eliminated from the treatment estimate by the balanced design, is also eliminated from the variance estimate. The reader is referred to *Cross-over Trials in Clinical Research*[25] for more details.

One particular advantage, however, of using mixed models, whether of the form represented by (1) or of the form represented by (7) (depending on the design employed) is that they permit estimation of the random effect variance, *ψ*^2^ This can then be used to produce so-called shrinkage estimates for future patients.

Suppose that we have established the values of T, the average treatment effect and *ψ*^2^ the variation of the treatment effect from patient to patient reasonably well on the basis of a series of n-of-1 trials. These can then be used to improve the estimate for a future patient, *i*. Suppose that this estimate is ${\hat{\tau }}_{i}$ and has been established based on observing the patient for *k* cycles. Its variance is then 2*σ*^2^/*k* By regarding T as a prior mean and *ψ*^2^ as a prior variance we can use the method of empirical Bayes to produce an improved estimator ${\hat{\tau }}_{i}^{*}$ This weights ‘prior’ mean and statistic by their respective precisions, that is to say the reciprocal of the variances.

We thus calculate

$${\hat{\tau }}_{i}^{*}=\frac{\frac{1}{\psi^{2}}T+\frac{k}{2\sigma^{2}}{\hat{\tau }}_{i}}{\frac{1}{\psi^{2}}+\frac{k}{2\sigma^{2}}}=\frac{2\sigma^{2}T+k\psi^{2}{\hat{\tau }}_{i}}{2\sigma^{2}+k\psi^{2}}$$ (8)

The variance of this estimate can be calculated using the standard formula *posterior precision = prior precision + data precision* (see for example, Miller and Miller [57]) and then that the posterior variance is the reciprocal of the posterior precision. We thus calculate

11ψ2+k(2σ2)=2σ2ψ22σ2+kψ2 (9)

If (8) and (9) are studied it can be shown that if *k* = 0, which corresponds to having no data on the current patient, then we simply use the overall mean T and the variance of our prediction on the true effect for the patient is *ψ*^2^. On the other hand, if either k or *ψ*^2^/*σ*^2^ is very large our estimate is close to ${\hat{\tau }}_{i}$ and has variance close to 2*σ*^2^/*k* In general, however, a weighted average ensues and the variance given by (9) is less than 2*σ*^2^/*k*.

Note that these formulae are approximate and treat the parameters of the ‘prior distribution’ as known. This is clearly not quite right. For example, the variance of the prediction, T, for a new patient for whom there are no data is assumed to be *ψ*^2^, representing the variance of the individual treatment effect from the true average over all patients. However, the prediction has to be issued using $\hat{T}$ and this *as an estimate of T* has a variance as given by (5) so that the variance of the prediction will be

$$\psi^{2}+\psi^{2}/n+2\sigma^{2}/\left(nk\right)$$ (10)

A further problem is the well-known one that affects all REML estimates; the nuisance parameters (in this instance *ψ*^2^,*σ*^2^) are not known[58]. Provided that the number of patients on which this ‘prior distribution’ is calculated is not very few, the approximations can nevertheless prove valuable. One of us (AA) is working on an improvement to estimation of the variance using the parametric bootstrap and this will form the basis of a further publication.

A good discussion is given by Cox and Solomon[59] pp55-56.

## Simulation

In this section we present the results of simulations to illustrate theoretical points made above. Unless stated otherwise, the model used for simulation is that given by Eq (1), although for some analyses this is unnecessarily complicated. For example, where the analysis reduces to within cycle differences, the parameter settings for pure cycle effects and hence also patient effects are irrelevant. Also, we shall mainly be examining variances and for this purpose average treatment effects are also irrelevant. In describing the simulation settings and the figures that accompany them we shall limit ourselves to mentioning those parameter settings that are relevant to make the point. We shall also assume a balanced case in which all patients have received both treatments in three cycles

**Fig 1:**
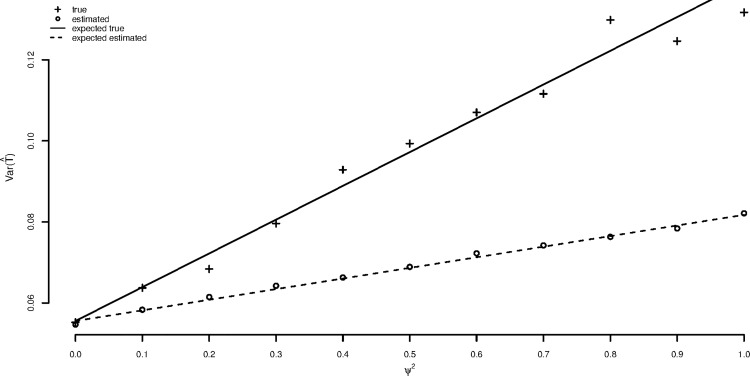
Comparison of true and estimated variances for the matched pairs approach (see text for explanation).

Fig 1 shows true and estimated variances of the treatment estimate for the matched pairs approach plotted against the variance *ψ*^2^ for the random treatment-by-patient interaction and this has been increased from a value of 0 to 1 in steps of 0.1. The within-cycle variance *σ*^2^ has been set to 1. One thousand trials have been simulated for each value of *ψ*^2^ The upper solid line is the theoretical true variance as given by formula (5) and the pluses are the corresponding values calculated as the variance of the 1000 estimates resulting from the simulation at each value of *ψ*^2^. The lower dashed line is the estimated variance using formula (6) and the circles are the corresponding values calculated as the means of the 1000 estimated variances of the estimate at each value of *ψ*^2^. It can be seen that simulation and theory agree.

The important feature is that as the random variance for the treatment-by-patient interaction increases, the degree by which the estimated variance for the matched pairs t underestimates the true variance increases. Only for the value of *ψ*^2^ = 0 do the two agree. As we have already discussed, this justifies using the matched pairs approach as a test of the strict null hypothesis that both treatments are identical. However, if the treatments are not identical then any element of personal response to treatment will lead to incorrect claimed coverage of confidence intervals.

**Fig 2:**
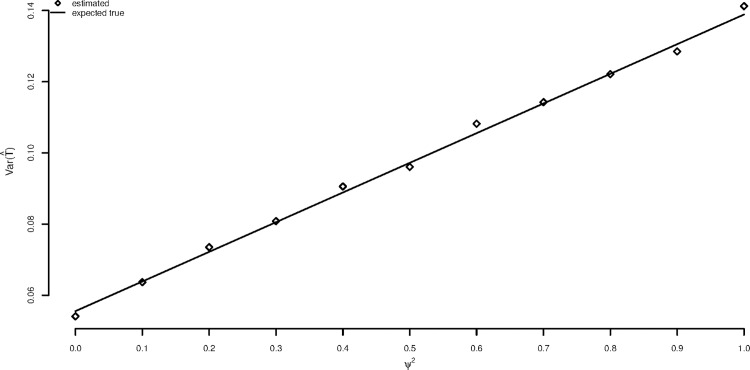
Comparison of estimated variances for the summary measures approach and the mixed model with the true variance.

Fig 2. plots the same true variance using Eq (5) as a solid line but now plots the simulated *estimated* variances for the summary measures approach (diamond). Again each point represents the result of 1000 runs. It can now be seen that both of these approaches produce estimated variances that are equivalent to the true one.

## An example

In this section we present a simulated example to illustrate the use of various approaches. We assume a series of n-of-1 trials in asthma for which two bronchodilators, A and B are given in a single dose with each period of treatment well separated by washout. We suppose that forced expiratory volume in one second (FEV_1_) has been measure in mL 12 hours after treatments and that 12 patients have been treated in three cycles each. That is to say the total number of periods in which patients have been treated is 6. The simulated data are shown in Table 6 below. These data are also available in Excel format as S1 Table in the supplementary information. Six of the values (two for patient 11 and four for patient 12) have been underlined and placed in italics. First all the data will be analysed since this gives a balanced case. Second, these six values will then be removed to illustrate the analysis in an unbalanced case.

**Table 6:** Simulated data from a trial in asthma. The data are for 12 patients treated in 3 cycles. The data are arranged in columns by treatment given. For each patient the first row represents the period in which the treatment was given and the second the result in ml of FEV1. Values in italics and underlined are those which are removed to create an unbalanced set.

	Treatment					
Patient	A	B	A	B	A	B
1	1	2	3	4	6	5
	2394	2686	2515	2675	2583	2802
2	2	1	3	4	6	5
	2746	2726	2592	2867	2743	2742
3	1	2	3	4	6	5
	2668	2560	2542	2584	2491	2737
4	1	2	3	4	6	5
	2397	2696	2411	2895	2499	2760
5	2	1	3	4	5	6
	3179	3221	2952	3096	2600	3192
6	1	2	4	3	5	6
	2643	2496	2759	2847	2651	2860
7	1	2	3	4	5	6
	2678	2843	2492	2763	2801	2890
8	2	1	3	4	5	6
	2887	2862	2875	3083	2689	2967
9	2	1	3	4	6	5
	2490	2841	2648	3044	2688	2914
10	2	1	3	4	6	5
	2268	2576	2413	2493	2344	2699
11	2	1	4	3	6	5
	2617	2923	2629	2832	*2732*	*2866*
					—-	—-
12	1	2	4	3	5	6
	2627	2759	*2712*	*2698*	*2572*	*2826*
			—-	—-	—-	—-

Analysis of the balanced case using a linear model in which effects for Patient, Cycle within Patient, Treatment-by-Patient in addition to Treatment are fitted produces an estimate of the residual variance of 11842 ml^2^. This is a within-patient variance pooled across all patients and not just calculated independently for each. The mean (naïve) treatment estimates can then be calculated for each patient. For any given patient these are based upon k = 3 pairs and so multiplying the variance of 11842 ml^2^ by 2/k gives the estimated variance of 2/3 × 11842 ml^2^ = 7895ml^2^ and hence a standard error of $\sqrt{7985ml^{2}}=88.9ml$ The results are given in *Table 7* where the number of cycles k (3 in every case) is given in column 2, the per patient naïve treatment estimate in column 3 and the standard errors in column 4. Since the pooled within patient variance is used to calculate the standard error and the number of observations is identically 3 for every patient, the standard error is identical for every patient.

**Table 7:** Naïve per patient treatment estimates and estimated standard errors for the 12 patients whose data are given in Table 6. The columns headed k give the number of observations per patient. See text for explanation.

	Balanced			Unbalanced		
Patient	K	Per patient estimates	Standard error	k	Per patient estimates	Standarderror
1	3	223.7	88.9	3	223.7	91.1
2	3	84.7	88.9	3	84.7	91.1
3	3	60.0	88.9	3	60.0	91.1
4	3	348.0	88.9	3	348.0	91.1
5	3	259.3	88.9	3	259.3	91.1
6	3	50.0	88.9	3	50.0	91.1
7	3	175.0	88.9	3	175.0	91.1
8	3	153.7	88.9	3	153.7	91.1
9	3	324.3	88.9	3	324.3	91.1
10	3	247.7	88.9	3	247.7	91.1
11	3	214.3	88.9	2	254.5	111.6
12	3	124.0	88.9	1	132.0	157.8

For the unbalanced case the number of cycles per patient are given in column 5. These are three as before for patients 1 to 10, for whom the estimates are identical but two for patient 11, for whom the estimate is now 254.5 ml and one for patient 12, for whom the estimate is now 132.0. To estimate standard errors we need to obtain the residual variance from the linear model as before but using the reduced data now available to us. Doing this we now obtain a slightly higher figure of 12446 ml2. This has to be first multiplied by two and then divided by three for patients 1 to 10 by two for patient 11 and by one for patient 12. The square root then gives us the values in column 7.

These estimates and standard errors can be put into a conventional meta-analysis routine. The fixed effects analysis will be appropriate if a test of the point null hypothesis that A is identical to B for every patient is undertaken and the random effects analysis if the patients are instead treated as a random sample from some wider population and it is desired either to produce an estimate of the mean effect for this population, with associated confidence interval, or it is desired to produce ‘shrunk’ improved estimates for the patients treated. Particularly convenient for this latter purpose is the metafor package in R of Viechtbauer[60]. In SAS® the macros developed by Senn et al may be used[61] (although the latter only produces the estimates and not their standard errors).

If a fixed effects analysis is carried out for the balanced and the unbalanced cases represented in Table 7, the estimates (standard errors) for the overall results are 188.7(25.6) and 194.5 (27.5) respectively. The corresponding random effects meta-analysis results using the DerSimonian & Laird approach are 188.7(28.4) and 194.5(29.6). All these analyses are clearly significant. In this case there is no difference between fixed and random effect estimates (this is inevitable for the balance case but not for the unbalanced one) but the standard errors are somewhat higher for the random effects meta-analysis.

If the metafor package is used the rma function may be used to carry out the meta-analysis and the blup function to produce shrunk estimates (BLUPs) and their standard errors. The rma function gives various options for the random effects approach and we have used the REML approach to produce the values in Table 8.

**Table 8:** Shrunk estimates and standard errors for a meta-analysis of the balanced case and unbalanced cases of Table 7 using the metafor package.

	Balanced		Unbalanced	
Patient	Estimate	SE	Estimate	SE
1	195.1	44.5	200.1	46.7
2	169.7	44.5	173.7	46.7
3	165.1	44.5	169.0	46.7
4	217.9	44.5	223.6	46.7
5	201.7	44.5	206.8	46.7
6	163.3	44.5	167.1	46.7
7	186.2	44.5	190.8	46.7
8	182.3	44.5	186.8	46.7
9	213.6	44.5	219.1	46.7
10	199.5	44.5	204.6	46.7
11	193.4	44.5	202.6	48.7
12	176.9	44.5	190.0	50.9

If *Table 7* is studied it can be seen that the standard errors are very much smaller than is the case for the naïve estimate. One should be a little cautious at taking these at face value since they are based on approximations. How one could do better would lead us into further complications that we do not pursue here but will reserve for a further more technical paper. Nevertheless, most of the reduction compared to the standard errors given in Table 7 is genuine and reflects the fact that in estimating treatment effects for a given patient we are able not only to use values from that patient but values from others. Since the variance of treatment by patient interaction is small in this example this bring about a considerable reduction.

**Fig 3:**
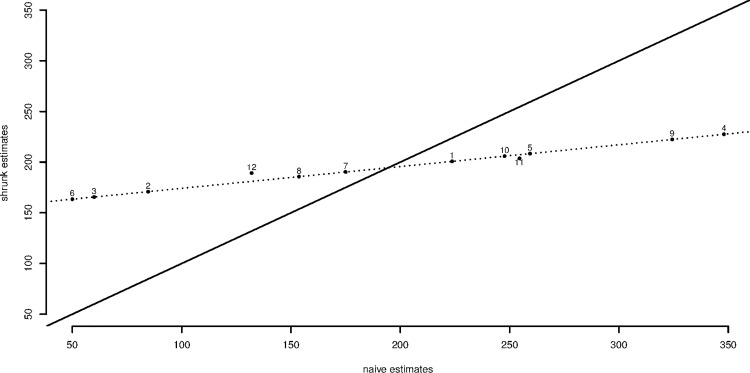
Shrunk and naïve estimates for 12 patients in the unbalanced case. The solid diagonal line is a line of equality. It can be seen that the estimates for individual patients are strongly shrunk and the dotted line gives the line of shrinkage for patients 1 to 10 who all have the same amount of information. The value for patient 11 is shrunk more strongly than for patients 1–10 because data from one cycle are missing. For patient 12 data from two cycles are missing and shrinkage is even stronger.

The second point is that the estimates have been strongly shrunk towards the mean. This is most easily seen by plotting the shrunk estimate against the naïve estimate as in Fig 3. To save space we reproduce the unbalanced case only as this is the more interesting one.

It is noticeable that for the 10 patients for whom three cycles of measurement are available the shrunk estimates, whilst much closer to the mean than the line of equality, all lie on a straight line. However, the estimates for patients 11 and 12 are subject to stronger shrinkage, since they are based on fewer measurements and thus are even closer to the overall mean.

The third point is that the standard errors for patients 11 and 12 for the unbalanced case are higher than for the other 10. This reflects the fact that less information is available for them. Nevertheless, most of the information available for any patient in this example is given by the overall mean and this is reflected in the fact that the standard errors for patients 11 and 12 in the unbalanced case are only slightly higher than they are for the other 10.

## Discussion

We have shown that where the randomisation has been carried out in cycles, then for the purpose of testing the strict null hypothesis that two treatments are identical, the matched-pairs approach proposed by Chen and Chen is valid. This is because under the null hypothesis that the treatments are identical it makes no difference which treatment any patient gets and hence the treatment-by-patient interaction is zero by hypothesis.

A further justification might be that even if one does not believe in the possibility of strict point null being true and one wishes to avoid what has been called a type III error, choosing the worse of two treatments, the fact that empirical evidence shows that large random effects are generally small where the average difference between treatments are small, justifies one in acting as if the random effects variance was zero at the dividing point of zero difference between the two treatments.

However, as soon as estimation is the object then one has to allow for the possibility of values that are in theory well away from zero. Thus for the purpose of calculating confidence intervals it may be wise to allow for a random patient by treatment interaction. In fact, even for the purpose of testing the strict null it may be worth allowing for an interaction as in the third column of *Table 3*. An analogy can be given with the common two sample t-test comparing two means, each based on *n* values. Under the null hypothesis the values in two samples have the same mean. Thus the most efficient estimate of the common variance *σ*^2^ is based on pooling *all* the values from *both* samples together, producing a sample variance with 2*n*-1 degrees of freedom. This would yield a valid test. The problem is that as the means start to differ, the variance estimate will start to increase and power will be affected. It is thus better to remove the one degree of freedom for the treatment estimate from the variance estimate and to estimate the variance separately *within* each treatment group to produce an overall estimate with 2*n*-2 degrees of freedom.

Returning to the case of analysing n-of-1 trials, the most powerful approach may be to remove the interaction from the estimate of the variance and test the null hypothesis using *n(k-1)* degrees of freedom. (There is an analogy to fixed effects meta-analysis here.) We make this recommendation with some hesitation. It may be useful for rare diseases where information is hard to get. However, for purposes other than hypothesis testing it cannot be recommended. For the purpose of producing confidence intervals for the mean treatment effect a simple valid analysis may be based on the summary measures approach. However, if it is desired to produce shrunk estimates, then either a mixed model approach such as given by our equations above or as produced by many statistical packages should be used.

A further advantage to mixed models is that for the unbalanced case they are more efficient than the summary measures approach. If some patients have been studied in fewer cycles than others, then the summary measures approach has the disadvantage that estimates that are less precise will be given the same weight as those that are more so. Optimal weighting requires simultaneous estimation of the treatment-by-patient variance and the within cycle variance and this is what the mixed model case achieves. Even more complicated is where cycles are incomplete so that only one treatment is given within a cycle and not two. This requires estimation of three components of variation.

We note that implementation of a mixed modelling approach may be difficult for many researchers. There are a number of complications that can arise and issues that need to be considered and we have decided in interests of space not to cover these in detail here but reserve them for a more technical exposition. A simple solution that will be easily available to many researchers is to use the summary measures approach we have illustrated here in connection with a meta-analysis package. The only difference from standard meta-analysis is that the variance of each estimate must be computed from a globally estimated variance rather than one using the local information only.

When the article was under peer review the referees rightly pointed to some limitations with what we have presented, which we feel are important to highlight here in the discussion.

The random effect model we have used to reflect possible differences in response to treatment is a smooth Normal mixture. That is to say that the differences from patient to patient from the average treatment effect are assumed to have a Normal distribution with expectation zero and unknown variance to be estimated. It is, of course, possible to imagine quite different models, for example, one in which there are a finite number of groups, each having a different response. Such a finite-mixture approach has occasionally been advocated in meta-analysis[62] and might be considered for n-of-1 trials.

We have proposed that identification of components of variance may be achieved through designs involving randomisation as a keystone of the approach. A possible alternative might be to rely on repeated measures *within* given treatment periods as being a means of identifying within-patient random variation and hence random treatment-by-patient interaction. Such an approach does require strong assumptions and is very much model-dependent. Nevertheless, this general approach has been used with success in the so-called ‘population pharmacokinetics’ field starting with the pioneering work of Lewis Sheiner and others[63] in the 1970s and might be considered in those cases where repeated randomisation is difficult.

The methods we have outlined are appropriate when the patients can be regarded as being exchangeable in advance of experimentation, that is to say that there is nothing apart from the results gained under treatment for a particular patient (and, of course, the average results we have from others) that can help us guide treatment for him or her. This is not the case if we can find covariates that are predictive of general response. Note that the main effect of patients is confounded with demographic covariates, so that adding covariates to the model brings nothing additional in this respect. However, this is not true of treatment-by-patient interaction, so using covariates might help identify the degree of response. This is promising in principle but it is beyond the scope of this paper.

Finally, we have considered continuous outcomes only and for random effect models used Normal-Normal mixtures. Other outcomes can be encountered in the n-of-1 setting and more widely, of course, in the case of cross-over trials. For example, Makubate and Senn[64] used Normal-logit mixtures for cross-over trials in infertility, some of which had repeated treatment periods. For count data, such as, for example, seizures in epilepsy or exacerbations in pulmonary disease, gamma-Poisson mixtures might be envisaged[65],

## Conclusion and Recommendations

Our recommendations are as follows.

For balanced n-of-1 designs randomised in cycles, a valid and powerful test of the strict null hypothesis that the treatments are identical may be performed using an analysis of variance with Patient/Cycle as the block structure and fitting a treatment-by-patient interaction.

For the purpose of producing confidence intervals and for the purpose of predicting the effects for future patients a random treatment-by-patient interaction should be allowed for and this is most easily done using a mixed model.

As an alternative to the design that randomises treatments within cycles in patients it may be useful to base designs on Latin squares balancing the distribution of treatments across periods and patients. Suitable analysis involves fitting patient effects as random, period effects as fixed and treatment-by-patient interactions as random.

A simple implementation of our general approach is by calculating summary measures for each patient and associated standard errors (with the assumption of homoscedasticity across patients) and using these as input to a meta-analysis. A fixed effects approach will provide a valid test of the strict null hypothesis of equality and a random effects approach will enable shrunk estimates to be produced.

We do not recommend attempting to adjust for carry-over but instead recommend using washout periods of adequate length. It should be noted that such periods do not have to be ones in which no treatment is given. The strategy of an *active washout* can be used[25], whereby patients can be switched immediately from one treatment to another (if safe to do so) but measurement starts once the effect of the previous treatment has disappeared and steady-state has been reached.

Our final point concerns reporting. A series of n-of-1 trials forms a complex data-set that needs to be reported in full and frank detail and a recent review by Li et al[66] of 112 studies involving 2,278 patients in total found that reporting was often poor. The recently created *CONSORT extension for reporting N-of-1 trials (CENT)*[67] statement would, if followed, do much to improve this and we recommend that authors use it when reporting trials.

## Supporting Information

S1 Table Data for the example in Excel format. *Patient* (1–12) is the patient number. *Cycle* (1–3) is the cycle within patient. *Pair* labels the 36 pairs of data by patient (first number) and cycle (second number). *Period* (1–6) is the period within patient. *Treatment* (A or B) is the treatment given. *FEV_1* is the outcome for forced expiratory volume in one second in mL. *Missing* is an indicator (1 = present, 2 = missing).
(XLS)
